# Dose Optimization of Apomorphine Sublingual Film for OFF Episodes in Parkinson’s Disease: Is the Prophylactic Use of an Antiemetic Necessary?

**DOI:** 10.3233/JPD-223537

**Published:** 2023-05-09

**Authors:** Robert A. Hauser, William G. Ondo, Yi Zhang, Alyssa Bowling, Bradford Navia, Eric Pappert, Stuart H. Isaacson, for the CTH-301 Study Investigators

**Affiliations:** a University of South Florida, Tampa, FL, USA; b Methodist Neurological Institute, Houston, TX, USA; c Sunovion Pharmaceuticals Inc., Marlborough, MA, USA; d Neurology Associates, San Antonio, TX, USA; e Parkinson’s Disease and Movement Disorders Center of Boca Raton, Boca Raton, FL, USA

**Keywords:** Antiemetics, apomorphine, domperidone, nausea, Parkinson’s disease, treatment, vomiting

## Abstract

**Background:**

Nausea is common upon initiating dopamine agonists in patients with Parkinson’s disease (PD); however, pretreatment with an antiemetic is recommended only when initiating apomorphine formulations.

**Objective:**

Evaluate the need for prophylactic antiemetic use during dose optimization of apomorphine sublingual film (SL-APO).

**Methods:**

A *post hoc* analysis of a Phase III study evaluated nausea and vomiting treatment-emergent adverse events in patients with PD who underwent SL-APO dose optimization (10–35 mg; 5-mg increments) to achieve a tolerable FULL ON. Frequencies of nausea and vomiting were described for patients who did versus did not use an antiemetic during dose optimization and by patient subgroups based on extrinsic and intrinsic factors.

**Results:**

Overall, 43.7% (196/449) of patients did not use an antiemetic during dose optimization; most of these patients (86.2% [169/196]) achieved an effective and tolerable SL-APO dose. In patients who did not use an antiemetic, nausea (12.2% [24/196]) and vomiting (0.5% [1/196]) were uncommon. An antiemetic was used in 56.3% (253/449) of patients, with 17.0% (43/253) and 2.4% (6/253) experiencing nausea and vomiting, respectively. All events of nausea (14.9% [67/449]) and vomiting (1.6% [7/449]) were of mild-to-moderate severity except for 1 event each. Irrespective of antiemetic use, among patients without baseline dopamine agonist use, nausea and vomiting rates were 25.2% (40/159) and 3.8% (6/159); in those already using dopamine agonists, rates were 9.3% (27/290) and 0.3% (1/290).

**Conclusion:**

Prophylactic treatment with an antiemetic is not necessary for most patients who initiate SL-APO for the treatment of OFF episodes in PD.

## INTRODUCTION

Gastrointestinal symptoms are common in patients with Parkinson’s disease (PD) [1, 2]. In addition, many PD medications can exacerbate these symptoms and cause nausea and vomiting, especially levodopa and dopamine agonists [3–5]. For non-apomorphine dopamine agonists (e.g., ropinirole, pramipexole, rotigotine), the incidence of nausea ranges from 11% to 60% and vomiting ranges from 4% to 20% [6–10]. For apomorphine, clinical trials in patients with PD have reported similar rates: subcutaneous injection (SC-APO), 6–73% (nausea or vomiting) [11–16]; sublingual film (SL-APO), 21–28% (nausea), 7% (vomiting) [17]; continuous infusion, 12–30% (nausea) [18, 19]; inhalation, 14–33% (nausea) [20].

Despite the common association of nausea and vomiting with dopamine agonist use, prophylactic antiemetics were not used in the clinical trials of non-apomorphine dopamine agonists in PD [21–27], and therefore, there is no recommendation for their use described in US Food and Drug Administration (FDA)-approved prescribing information [6–10]. However, key trials of all apomorphine formulations used prophylactic antiemetics [11, 16, 17, 20, 28–30]; therefore antiemetics, either trimethobenzamide or domperidone, depending on the approval status in the relevant country/region, are recommended in prescribing information [31–33].

Based on the pivotal trial, the US prescribing information for SL-APO recommends prophylactic use of trimethobenzamide 300 mg 3 times a day, beginning 3 days prior to the initial dose [33]; however, trimethobenzamide has been in short supply or unavailable and domperidone is not approved for use in the US [34]. Despite the historic use of antiemetic agents with rapid-acting apomorphine formulations, the prophylactic benefit imparted by trimethobenzamide and domperidone has appeared modest and therefore may not be necessary in many cases for successful initiation of apomorphine preparations [17, 35–39]. Herein, we report data on dose optimization of SL-APO without antiemetic use, based on data from a Phase III trial.

## MATERIALS AND METHODS

This *post hoc* analysis evaluated data from an open-label, Phase III, long-term safety and efficacy study initiated in August 2015 (ongoing; NCT02542696) with an interim analysis data cutoff date of September 2020. The study was designed, conducted, and monitored in accordance with the World Medical Association Declaration of Helsinki (1989) and International Council for Harmonisation guidelines. An institutional review board, research ethics board, or independent ethics committee approved the study protocol and patient informed consent forms.

## Patients

Eligible patients could be new (no prior exposure to SL-APO) or rollover patients from prior SL-APO studies (CTH-201 [NCT03187301]: placebo- and positive-control crossover study; CTH-203 [NCT03292016]: comparative bioavailability study; CTH-300 [NCT02469090]: placebo-controlled study; or CTH-302 [NCT03391882]: comparative crossover study). Key eligibility criteria are provided in Supplementary Table 1. Briefly, patients were eligible if they had idiopathic PD by UK Brain Bank criteria, were responsive to and receiving stable doses of carbidopa/levodopa with or without adjunctive PD medications, and experienced ≥1 OFF episode per day with ≥2 h of daily OFF time. Key exclusion criteria included atypical or secondary parkinsonism; clinically significant oral pathology within 30 days of screening; or medical, surgical, psychiatric, or laboratory abnormalities judged to be clinically significant by the investigator.

## Study design

SL-APO treatment was initiated with an open-label dose-optimization phase to determine the patient’s effective and tolerable dose (Supplementary Figure 1). Dose optimization occurred during sequential office visits when the patient was OFF to determine the individualized dose of SL-APO (10–35 mg; 5-mg increments) that resulted in a tolerable FULL ON within 45 min. FULL ON was achieved when SL-APO treatment provided the patient with benefit related to mobility, stiffness, and slowness; adequate motor function to perform normal daily activities; and a response that was comparable to or better than the normal response to their PD medications before enrolling in the study. After a tolerable FULL ON dose was identified, patients progressed into an open-label maintenance phase. Initially, rollover patients from a prior SL-APO study were optimized to SL-APO as described above. Following a protocol amendment, rollover patients were assigned the optimized dose of SL-APO that was administered in the previous study. The duration of time between studies varied for rollover patients.

Initially, the protocol mandated treatment with a prophylactic antiemetic (trimethobenzamide 300 mg TID in the US or domperidone 10 mg twice daily outside the US) beginning 3 days before initiation of dose optimization, which was to be continued through the end of this phase. Following a protocol amendment, prophylactic antiemetic use was made optional to be able to evaluate the need for an antiemetic during dose optimization; use of an antiemetic was allowed only if clinically warranted as per the investigator’s discretion. Safety assessments for treatment-emergent adverse events (TEAEs) were collected at every visit. Serious TEAEs were defined as any untoward medical occurrence that resulted in death, was life-threatening, required hospitalization or prolongation of existing hospitalization, resulted in permanent disability/incapacity, was a congenital anomaly, or was an important medicalevent.

## Statistical analysis

Results were summarized descriptively for the dose-optimization phase population (all patients who enrolled in the study and received ≥1 dose of study medication during the dose-optimization phase) and included the incidence, maximum severity, and discontinuations due to nausea and vomiting TEAEs, as well as incidence of nausea and vomiting by SL-APO dose. The incidences of nausea and vomiting were also descriptively evaluated according to a variety of extrinsic and intrinsic factors. Extrinsic factors included study enrollment status (new versus rollover patients); use of an antiemetic (domperidone or trimethobenzamide) at any time during dose optimization; use of other concomitant dopamine agonists, monoamine oxidase-B (MAO-B) inhibitors, amantadine, anticholinergics, or antidepressants at baseline (i.e., prior to the first dose of SL-APO during dose optimization); baseline daily levodopa dose (<500, ≥500 to <900, and ≥900 mg); and smoking status (current, never, and former). Overall, TEAEs were reported descriptively for the full dose-optimization population and as a function of antiemetic use. Intrinsic factors included sex (male versus female), age (<65 versus ≥65 years), weight (<80 versus ≥80 kg), region (US versus outside US), time since diagnosis of PD (<8 versus ≥8 years), time since diagnosis of motor fluctuations (<3 years versus ≥3 years), Hoehn and Yahr stage (<2.5 versus ≥2.5), and number of daily OFF episodes.

A further evaluation of nausea and vomiting TEAEs experienced during SL-APO dose optimization was conducted in newly enrolled patients after the protocol amendment in which prophylactic antiemetic use was made optional.

## RESULTS

### Patients and demographics

The dose-optimization phase population included 449 patients. The majority were male (66.4%), White (96.0%), from the US (87.1%), and had a mean (SD) age of 64.3 (8.8) years (Table 1). Overall, 82.2% were new patients with no prior exposure to SL-APO. All patients were receiving carbidopa/levodopa. Dopamine agonists were used concomitantly at the time of the first dose of SL-APO by 60.4% of patients, with numerically lower dopamine agonist use among patients from the US (56.5%) versus outside the US (86.2% Supplementary Table 2). A subset of the study population was analyzed to specifically assess nausea and vomiting rates in new patients who enrolled in the study and underwent dose optimization after the protocol was amended to make the use of prophylactic antiemetics optional as opposed to mandatory. Baseline characteristics of this population (*n* = 188) were similar to those of the overall population, with the exception that all new patients enrolled after the protocol amendment were from the US (Supplementary Table 3).

**Table 1:** Demographics and baseline disease

Characteristic	Dose-Optimization
	Phase^a^ (N = 449)
Age, y, mean (SD)	64.3 (8.8)
Male, *n* (%)	298 (66.4)
Race, *n* (%)
White	431 (96.0)
Black or African American	10 (2.2)
Other	8 (1.8)
Weight, kg, mean (SD)	82.1 (18.5)
Region, *n* (%)
US	391 (87.1)
Outside US	58 (12.9)
Enrollment status
New^b^	369 (82.2)
Rollover^c^	80 (17.8)
Time since PD diagnosis, y, mean (SD)^d^	8.6 (4.5)
Time since motor fluctuations, y, mean (SD)^d^	4.5 (3.8)
Modified Hoehn and Yahr stage when ON, *n* (%)
0	2 (0.4)
1 or 1.5	21 (4.7)
2 or 2.5	296 (65.9)
3	42 (9.4)
4	1 (0.2)
Missing	87 (19.4)
Number of OFF episodes per day, mean (SD)^d^	3.9 (1.3)
Total daily levodopa dose, mg, median	900.0
Any antiemetics use, *n* (%)^e^	253 (56.3)
Domperidone	43 (17.0)
Trimethobenzamide	207 (81.8)
Domperidone/trimethobenzamide^f^	3 (1.2)
Concomitant PD medications, *n* (%)^g^
Dopamine agonists	271 (60.4)
MAO-B inhibitors	202 (45.0)
Amantadine	100 (22.3)

^a^Dose-optimization population (all patients who enrolled in the study and received ≥1 dose of study medication during the dose-optimization phase); ^b^New patients had no prior exposure to SL-APO; ^c^Rollover patients had completed a prior SL-APO study (NCT03187301, NCT03292016, NCT02469090, or NCT03391882); ^d^Safety population (N = 467; all patients who enrolled in the study and received ≥1 dose of study medication); ^e^Patients used an antiemetic during dose optimization; ^f^Patients used domperidone and trimethobenzamide during dose optimization, but details regarding timing of use for each agent is unknown; ^g^Defined as medication use overlapping with the first dose of SL-APO during dose optimization. MAO-B, monoamine oxidase-B; PD, Parkinson’s disease; SL-APO, apomorphine sublingual film.

**Table 2:** Characteristics of patients who did not use an antiemetic at any time and achieved an effective and tolerable dose of SL-APO during dose optimization

Parameter, *n* (%)	Did Not Use an Antiemetic and Achieved Successful Dose Optimization (N = 169)
SL-APO dose
10 mg	47 (27.8)
15 mg	41 (24.3)
20 mg	39 (23.1)
25 mg	24 (14.2)
30 mg	12 (7.1)
35 mg	6 (3.6)
Concomitant dopamine agonist use^a^
Yes	107 (63.3)
No	62 (36.7)
Baseline daily levodopa dose
<500 mg	24 (14.2)
≥500 to <900 mg	64 (37.9)
≥900 mg	74 (43.8)
Missing	7 (4.1)
Enrollment status
New^b^	136 (80.5)
Rollover^c^	33 (19.5)

^a^Defined as medication use overlapping with the first dose of SL-APO during dose optimization; ^b^New patients had no prior exposure to SL-APO; ^c^Rollover patients had completed a prior SL-APO study (NCT03187301, NCT03292016, NCT02469090, or NCT03391882). SL-APO, apomorphine sublingual film.

**Table 3:** Summary of nausea or vomiting TEAEs in the overall population (N = 449)^a^

Parameter, *n* (%)	Nausea	Vomiting
Overall	67 (14.9)	7 (1.6)
Maximum severity of events
Mild	47 (70.1)	3 (42.9)
Moderate	19 (28.4)	3 (42.9)
Severe	1 (1.5)	1 (14.3)
Serious events	0	0
Events by SL-APO dose^b^
10 mg	28 (41.8)	2 (28.6)
15 mg	20 (29.9)	2 (28.6)
20 mg	11 (16.4)	1 (14.3)
25 mg	6 (9.0)	0
30 mg	8 (11.9)	1 (14.3)
35 mg	2 (3.0)	0
Events leading to discontinuation	8 (1.8)	0

^a^Dose-optimization population (all patients who enrolled in the study and received ≥1 dose of study medication during the dose-optimization phase); ^b^Patients may appear in multiple dose groups. SL-APO, apomorphine sublingual film; TEAE, treatment-emergent adverse event.

### Outcome of dose optimization and antiemetic use

Of 449 patients (US, 87.1%; outside US, 12.9%) who underwent SL-APO dose optimization, 88.4% (*n* = 397) successfully achieved an effective and tolerable dose. An antiemetic was used by 253 (56.3%) patients at some point during dose optimization. Of these patients, most used trimethobenzamide (81.8%), with fewer patients using domperidone (17.0%). Three (1.2%) used both antiemetics at some time during dose optimization. Data collection methods did not distinguish between prophylactic versus reactive antiemetic use.

There were 196 (43.7%) patients who did not use an antiemetic at any time during dose optimization. Of these, 154/196 (78.6%) were new patients (non-rollover) who enrolled after the protocol amendment, 29/196 (14.8%) were rollover patients who enrolled after the protocol amendment, and 13/196 (6.6%) were patients who enrolled before the protocol amendment (6 new patients and 7 rollovers). Of the 196 patients who did not use an antiemetic at any time during dose optimization, 169 (86.2%) achieved an effective and tolerable dose of SL-APO. The characteristics of this population were similar to the overall population. The majority (75.1%; 127/169) reached their effective and tolerable dose of SL-APO within the first 3 doses (10–20 mg; Table 2). Most patients were newly enrolled (80.5%); at baseline, most were using concomitant dopamine agonists (63.3%) and were taking ≥500 mg of levodopa (81.7%).

### Overall experience of nausea and vomiting

Overall, nausea occurred in 14.9% (67/449) and vomiting occurred in 1.6% (7/449) of patients during dose optimization (Table 3). For the 67 patients who did experience nausea, events were mild to moderate in 98.5% (*n* = 66) of patients and severe in 1.5% (*n* = 1). For the 7 patients who experienced vomiting, the severity was mild to moderate in 85.7% (*n* = 6) of patients and severe in 14.3% (*n* = 1). No serious TEAEs of nausea or vomiting were observed. Discontinuations due to nausea occurred in 1.8% of all patients, and there were no events of vomiting that led to discontinuation.

Of 397 patients who successfully achieved an effective and tolerable dose of SL-APO, nausea was experienced by 12.3% (*n* = 49) of patients and vomiting was experienced by 1.3% (*n* = 5) of patients during dose optimization. Among those who experienced nausea or vomiting during dose optimization and continued into the long-term maintenance phase, nausea occurred in 44.9% (22/49) of patients and vomiting occurred in 40.0% (2/5) of patients during maintenance treatment. Among those who did not experience nausea or vomiting during dose optimization, 19.1% (66/346) went on to report nausea or vomiting at some point during maintenance treatment.

### Nausea and vomiting by extrinsic and intrinsic factors

The incidences of nausea and vomiting were similar in patients who enrolled as new patients (nausea, 15.2%; vomiting, 1.6%) and in those who rolled over from a previous SL-APO study (nausea, 13.8%; vomiting, 1.3%; Table 4). In the population who used an antiemetic at any time during dose optimization, nausea occurred in 17.0% (43/253) of patients and vomiting occurred in 2.4% (6/253) of patients. In patients who did not use any antiemetic, the incidence of nausea was 12.2% (24/196) and the incidence of vomiting was 0.5% (1/196). The incidences of nausea were 11.6% in patients who used domperidone and 18.4% in patients who used trimethobenzamide. Vomiting was reported in 0 patients who used domperidone and in 2.9% of those who used trimethobenzamide. Three patients used both trimethobenzamide and domperidone at some point during the dose-optimization phase; no nausea or vomiting TEAEs were reported for these patients.

**Table 4:** Incidence of nausea or vomiting in comparisons of interest^a^

Parameter, % (*n*/N^b^)	Nausea	Vomiting
Overall	14.9 (67/449)	1.6 (7/449)
**Extrinsic factors**
Enrollment status
New^c^	15.2 (56/369)	1.6 (6/369)
Rollover^d^	13.8 (11/80)	1.3 (1/80)
Antiemetic use at any time^e^
Yes	17.0 (43/253)	2.4 (6/253)
Domperidone	11.6 (5/43)	0 (0/43)
Trimethobenzamide	18.4 (38/207)	2.9 (6/207)
Domperidone/trimethobenzamide^f^	0 (0/3)	0 (0/3)
No	12.2 (24/196)	0.5 (1/196)
Dopamine agonist use at baseline^g^
Yes	9.3 (27/290)	0.3 (1/290)
No	25.2 (40/159)	3.8 (6/159)
MAO-B inhibitor use at baseline^g^
Yes	17.7 (37/209)	1.0 (2/209)
No	12.5 (30/240)	2.1 (5/240)
Amantadine use at baseline^g^
Yes	10.3 (11/107)	0 (0/107)
No	16.4 (56/342)	2.0 (7/342)
Antidepressant use at baseline^g^
Yes	0 (0/7)	0 (0/7)
No	15.2 (67/442)	1.6 (7/442)
Anticholinergic use at baseline^g^
Yes	30.0 (3/10)	0 (0/10)
No	14.6 (64/439)	1.6 (7/439)
Baseline daily levodopa dose, mg
<500	12.5 (7/56)	5.4 (3/56)
≥500 to <900	16.8 (26/155)	1.9 (3/155)
≥900	14.3 (32/223)	0.4 (1/223)
Smoking status
Current	8.7 (2/23)	0 (0/23)
Never	15.2 (46/302)	2.0 (6/302)
Former	15.3 (19/124)	0.8 (1/124)
**Intrinsic factors**
Sex
Male	13.8 (41/298)	0.7 (2/298)
Female	17.2 (26/151)	3.3 (5/151)
Age, y
<65	13.6 (30/221)	1.8 (4/221)
≥65	16.2 (37/228)	1.3 (3/228)
Weight, kg
<80	14.7 (32/217)	1.4 (3/217)
≥80	15.3 (35/229)	1.7 (4/229)
Region
US	15.6 (61/391)	1.8 (7/391)
Outside US	10.3 (6/58)	0 (0/58)
Time since diagnosis of PD, y
<8	16.8 (35/208)	2.4 (5/208)
≥8	13.3 (32/241)	0.8 (2/241)
Time since diagnosis of motor fluctuations, y
<3	13.5 (23/170)	2.9 (5/170)
≥3	15.5 (43/277)	0.7 (2/277)
Hoehn and Yahr stage
<2.5	16.2 (43/266)	1.9 (5/266)
≥2.5	12.5 (12/96)	1.0 (1/96)
Number of OFF episodes per day
1	60.0 (3/5)	0 (0/5)
2	16.2 (6/37)	0 (0/37)
3	14.2 (18/127)	1.6 (2/127)
4	10.3 (16/156)	1.3 (2/156)
5	19.7 (15/76)	2.6 (2/76)
≥6	19.6 (9/46)	2.2 (1/46)

^a^Dose-optimization population (all patients who enrolled in the study and received ≥1 dose of study medication during the dose-optimization phase); ^b^N is the total number of patients in each factor subgroup; ^c^New patients had no prior exposure to SL-APO; ^d^Rollover patients had completed a prior SL-APO study (NCT03187301, NCT03292016, NCT02469090, or NCT03391882); ^e^Defined as use of an antiemetic (domperidone or trimethobenzamide) at any time during dose optimization; ^f^Patients used domperidone and trimethobenzamide during dose optimization, but details regarding timing of use for each agent are unknown; ^g^Defined as use prior to the first dose of SL-APO during dose optimization. MAO-B, monoamine oxidase-B; PD, Parkinson’s disease; SL-APO, apomorphine sublingual film.

**Table 5:** Summary of nausea and vomiting TEAEs in new patients enrolled after prophylactic antiemetic use was made optional and who did and did not use an antiemetic^a^

Parameter, *n* (%)	Used an Antiemetic^b^	Did Not Use an Antiemetic^b^	Overall
	(*n* = 34)	(*n* = 154)	(*n* = 188)
Nausea	17 (50.0)	21 (13.6)	38 (20.2)
Maximum severity of nausea
Mild	11 (64.7)	16 (76.2)	27 (71.1)
Moderate	5 (29.4)	5 (23.8)	10 (26.3)
Severe	1 (5.9)	0	1 (2.6)
Nausea leading to discontinuation	3 (17.6)	0	3 (7.9)
Vomiting	4 (11.8)	1 (0.6)	5 (2.7)
Maximum severity of vomiting
Mild	1 (25.0)	1 (100.0)	2 (40.0)
Moderate	2 (50.0)	0	2 (40.0)
Severe	1 (25.0)	0	1 (20.0)
Vomiting leading to discontinuation	0	0	0

^a^Safety population (all patients who enrolled in the study and received ≥1 dose of study medication); ^b^Population was comprised entirely of US patients and therefore trimethobenzamide was the only antiemetic available for use. TEAE, treatment-emergent adverse event.

The association between other evaluated drugs used at baseline and smoking status and the incidences of nausea and vomiting varied (Table 4). Among patients who used dopamine agonists, the incidence of nausea (9.3%) and vomiting (0.3%) were less than those who did not use dopamine agonists (nausea, 25.2%; vomiting, 3.8%). Numerically higher incidences of nausea were observed for patients who used MAO-B inhibitors (17.7%) versus those who did not (12.5%); there were no notable trends in vomiting rates. Numerically lower incidences of nausea were observed for patients who used amantadine (10.3%) versus those who did not (16.4%), but rates of vomiting were similar. Daily levodopa dose did not impact nausea or vomiting rates. Finally, current smokers had a numerically lower incidence of nausea (8.7%) compared with never (15.2%) or former smokers (15.3%), whereas vomiting rates were generally comparable.

Numerically higher incidences of nausea and vomiting were observed in women versus men (nausea, 17.2% versus 13.8%; vomiting, 3.3% versus 0.7%; Table 4). The incidence of nausea was numerically higher in patients in the US (15.6%) versus outside the US (10.3%), in patients with a shorter time since diagnosis of PD (<8 years: 16.8% vs ≥8 years: 13.3%), and in those with a lower Hoehn and Yahr stage (<2.5: 16.2% vs ≥2.5: 12.5%). The incidences of vomiting were similar for these subgroups.

### Experience of nausea and vomiting in new patients enrolled after prophylactic antiemetic use was made optional

In the population of new patients who enrolled in the study and underwent dose optimization after the protocol was amended to make the use of prophylactic antiemetics optional (*n* = 188), 81.9% (*n* = 154) of patients did not use an antiemetic (specifically trimethobenzamide) and 13.6% (21/154) of these patients experienced nausea (Table 5). The incidence of vomiting was 0.6% (1/154). There were no discontinuations due to nausea or vomiting in patients who did not use trimethobenzamide. The severities of nausea and vomiting TEAEs were similar to the overall population. Of those who did not use trimethobenzamide, 85.7% (132/154) successfully achieved an effective and tolerable dose of SL-APO.

In this population of 188 patients, 18.1% (*n* = 34) used trimethobenzamide during dose optimization based on investigator discretion. Investigator rationale for deciding to use trimethobenzamide was not recorded and it could not be determined if antiemetic use was reactive, in response to nausea or another adverse event or symptom, or prophylactic. In patients who did use trimethobenzamide, the incidence of nausea was 50.0% (17/34) and the incidence of vomiting was 11.8% (4/34). Three (17.6%) of 17 patients who used trimethobenzamide and experienced nausea, discontinued because ofnausea.

### Other safety findings

The incidence of any TEAE was 51.7% (232/449) in all patients during the dose-optimization phase, 56.5% (143/253) in patients who used an antiemetic, and 45.4% (89/196) in patients who did not use an antiemetic (Supplementary Table 4). Numerically higher incidences of fatigue, headache, hypertension, oral mucosal erythema, and yawning were observed in patients who used an antiemetic versus those who did not. The incidences of dizziness, orthostatic hypertension, and somnolence were mostly similar, and the incidence of dyskinesia was numerically lower in patients who used versus did not use an antiemetic.

## DISCUSSION

Gastrointestinal symptoms, including nausea and vomiting, are common in patients with PD and treatment with dopaminergic therapies can introduce or exacerbate these symptoms [3–5]. However, prophylactic antiemetic treatment is not recommended for non-apomorphine dopamine agonists [6–10], but it is recommended for apomorphine formulations [32, 33]. In this *post hoc* analysis of data from the dose-optimization phase of an open-label, long-term safety and efficacy study of SL-APO, rates of nausea and vomiting were similar to those for non-apomorphine dopamine agonists [6–10] and other apomorphine preparations, including SC-APO [11–16], continuous apomorphine infusion [18, 19], and inhaled apomorphine [20]. Severe TEAEs of nausea and vomiting were relatively infrequent during SL-APO dose optimization and few patients discontinued during this phase of the study because of nausea. These observations are consistent with findings reported for SC-APO, in which most events of nausea and/or vomiting were reported to be of mild to moderate severity and discontinuations due to nausea occurred in only ∼4–6% of patients [11, 13, 37, 40], and for continuous apomorphine infusions, in which ≈3% of patients discontinued owing to gastrointestinal complications [41]. Importantly, less than half of all patients in the current analysis who experienced nausea and vomiting went on to experience these events again during maintenance treatment, suggesting that patients can develop tolerance to these side effects over time.

The results of this analysis suggest that prophylactic use of an antiemetic is not necessary for most patients during dose optimization of SL-APO for OFF episodes in PD. Most (86.2%) patients who did not use an antiemetic successfully achieved an effective and tolerable dose of SL-APO. This rate was numerically higher than that reported from a study of SC-APO in which 63.0% of patients completed dose optimization without trimethobenzamide [37]. This prior SC-APO study and others corroborate the findings of our analysis that question the benefit of prophylactic use of an antiemetic [36–38]. Patients optimized on SC-APO without a prophylactic antiemetic demonstrated comparable rates of nausea and vomiting to those described for other dopamine agonists [36] and pretreatment with trimethobenzamide beginning 3 days prior to the first dose demonstrated no or only minimal benefit in the reduction of nausea and/or vomiting rates [37]. Further, another study found that nausea was more common in patients treated with trimethobenzamide 3 days before SC-APO versus patients without pretreatment, and the authors postulated this effect could have been due to acclimation to the antinausea effects of trimethobenzamide, that trimethobenzamide may be more effective as a single dose, or that there was simply no benefit to pretreatment [38].

The incidences of nausea and vomiting during SL-APO dose optimization were numerically lower in patients concurrently taking other dopamine agonists at baseline, consistent with findings reported in the literature for SC-APO in which the rate of nausea and/or vomiting was numerically lower in patients who used concomitant dopamine agonists at baseline versus those who did not [13, 37]. Rates of nausea and vomiting observed in this study in newly enrolled patients with a lack of prior exposure to SL-APO were comparable to those among rollover patients with prior SL-APO exposure. It is reasonable to assume that those with prior SL-APO exposure may have had tolerance to nausea and vomiting events. However, SL-APO is a rapid-acting therapy taken when needed; therefore, any protective effect of prior exposure may have been less pronounced than that experienced from continuous, daily exposure to non-apomorphine dopamine agonists. In addition, the duration of time between studies for rollover patients was variable; for those with a longer duration of time in between studies, any benefit of prior exposure may have waned.

The present analysis demonstrated numerically higher incidences of nausea and vomiting in patients from the US versus those from outside the US. The rationale for this finding may be multifactorial. Concomitant dopamine agonist use at baseline was much greater in patients from outside the US versus those in the US and the review of extrinsic and intrinsic factors suggested that dopamine agonist use mitigated rates of nausea and vomiting. In addition, a numerically higher incidence of nausea and vomiting was observed during SL-APO dose optimization in patients treated with trimethobenzamide (utilized in the US) versus domperidone (utilized outside the US).

Although antiemetics can be prescribed prophylactically to potentially lessen the occurrence of nausea and vomiting, their use should be carefully considered in the context of cost, the burden on patients to take additional medications multiple times daily, and limited access, at least within the US [34, 37, 42, 43]. Considering the shortage of trimethobenzamide in the US and the regional differences discussed previously, it was important to understand the potential benefit of antiemetic use, which was investigated in the subgroup of new (non-rollover) patients enrolled after prophylactic treatment with an antiemetic was made optional. In this subgroup, comprised entirely of US patients, the incidence, severity, and discontinuations due to nausea and vomiting TEAEs largely mirrored the overall population. Additionally, most patients (85.7%) who did not use an antiemetic (specifically trimethobenzamide) were able to successfully achieve an effective and tolerable dose of SL-APO. These data suggest that prophylactic trimethobenzamide may not be necessary for SL-APO dose optimization.

This analysis also suggested that the following patient subgroups may have a numerically lower risk for nausea and vomiting: male patients, current smokers, patients with a longer duration of PD (≥8 years), and those who had a Hoehn and Yahr stage ≥2.5. The observed numerically higher rates of nausea and vomiting in female versus male patients was similar to a study evaluating the frequency of nonmotor symptoms in patients with PD [44]. Furthermore, postoperative nausea and vomiting is 2–4 times more likely in female than male patients [45]. Numerically lower rates of nausea and vomiting in current smokers compared with former smokers or those who never smoked may be related to the antiemetic effects of nicotine [46]. Numerically lower rates of nausea and vomiting in patients with time since diagnosis of PD ≥8 years or who had a Hoehn and Yahr stage ≥2.5 may be related to potential tolerance to dopaminergic therapies in these patients with longer duration or more severe disease. No other factors were found to affect nausea and vomiting following treatment. Additional studies are required to further investigate demographic and clinical features that make a patient more susceptible to apomorphine-induced gastrointestinal upset.

Although these data suggest that prophylactic use of an antiemetic may not be necessary to successfully identify the optimal dose of SL-APO in most patients, there may be patients for whom an antiemetic demonstrates benefit. Therefore, the choice to use or not use an antiemetic should be evaluated in the context of anticipated risks and benefits. Antiemetics other than domperidone and trimethobenzamide could conceivably be utilized if necessary, but alternatives should be carefully considered because of contraindications or safety concerns. Antiemetics of the 5-hydroxytryptamine type 3 antagonist class (e.g., ondansetron, granisetron) are contraindicated with apomorphine owing to reports of loss of consciousness and profound hypotension [32, 33, 47]. In a clinical study, 3 of 12 healthy volunteers experienced serious adverse reactions, including severe hypotension, syncope/loss of consciousness, and bradycardia, and 1 healthy volunteer experienced seizures following concomitant administration of an investigational dissolving tablet formulation of apomorphine with ondansetron [48]. Central dopaminergic antiemetics (e.g., metoclopramide, prochlorperazine) may induce parkinsonism and antagonize the effect of dopaminergic therapies [47]. Other agents (e.g., naloxone, propofol) have been considered but have failed to gain more widespread use as antiemetics[49, 50].

There were several limitations of the current *post hoc* analysis. Evaluation of the role of antiemetics, concomitant dopamine agonists, or other extrinsic/intrinsic factors on the incidence of nausea and vomiting was not prespecified in the prospective Phase III study. After prophylactic antiemetic use was made optional per study protocol, an antiemetic may have been prescribed in reaction to events of nausea and vomiting, and therefore the impact of complete prohibition of antiemetics is unknown. Diaries collecting data on daily antiemetic use were not used; therefore, it was not possible to validate whether an antiemetic was used at the prescribed dosage, frequency, or duration, and it was not possible to definitively identify if patients took an antiemetic prophylactically (to prevent nausea/vomiting) or reactively (to address an event of nausea/vomiting). Likewise, it was not possible to track the continued use of concomitant dopamine agonists and other nonstudy medications to better understand any possible association with the incidence of nausea or vomiting. The study enrolled both new patients with no prior SL-APO exposure and rollover patients having previously received treatment; most analyses did not evaluate these populations separately. Further, while all patients who rolled over from prior studies underwent SL-APO dose optimization, patients who were previously enrolled in the pivotal study may have received placebo during the double-blind treatment phase and were not analyzed independently of those who received SL-APO during that phase. As previously noted, given the intermittent nature of SL-APO and considering that duration of time between studies was variable for rollover patients, it is unlikely that prior exposure to SL-APO in this small subset of patients impacted the results. The incidence of nausea and, particularly, vomiting was small overall; therefore, any comparisons that were undertaken with small sample sizes should be considered with caution.

These findings represent the largest analysis of the experience of nausea and vomiting associated with the use of apomorphine in a population of patients with PD and OFF episodes. A broad array of subpopulations of interest and other intrinsic and extrinsic factors were evaluated for their association with nausea and vomiting. Notwithstanding the limitations mentioned previously, these findings may inform treatment approaches and strategies when choosing to use SL-APO to manage OFF episodes.

## Conclusions

In this safety and efficacy study, most patients who did not use an antiemetic achieved an effective and tolerable dose of SL-APO. The overall experience of nausea and vomiting associated with SL-APO was generally consistent with what would be expected for other dopamine agonists. Events of nausea and vomiting were predominantly mild to moderate in severity and infrequently led to discontinuation, and most patients who experienced nausea or vomiting during dose optimization did not go on to report it again with longer-term treatment. Nausea and vomiting rates were numerically lower among patients who used versus did not use concomitant dopamine agonists at baseline, outlining a patient population that may be at lower overall risk for nausea and vomiting events. Overall, this analysis suggests that prophylactic use of an antiemetic may not be necessary for most patients to achieve successful dose optimization of SL-APO for the treatment of OFF episodes.

## Supplementary Material

Supplementary Material

## Prior publication

Presented in part at the International Parkinson and Movement Disorder Society (MDS) Virtual Congress 2020; September 12–16, 2020; the International Association of Parkinson’s & Related Disorders Society (IAPRD) Virtual World Congress, May 1–4, 2021; the International Parkinson and Movement Disorder Society (MDS) Virtual Congress 2021; September 17–22, 2021; the International Association of Parkinson’s & Related Disorders Society (IAPRD) XXVII World Congress, May 1–4, 2022; and the 4th Pan American Parkinson’s Disease and Movement Disorders Congress (MDS-PAS); May 26–28, 2022.
