# Supplementary material for: Dose Optimization of Apomorphine Sublingual Film for OFF Episodes in Parkinson’s Disease: Is the Prophylactic Use of an Antiemetic Necessary?
Source: J Parkinsons Dis. 2023 May 9;13(3):403–14. doi: 10.3233/JPD-223537 (PMC10200146; doi:10.3233/JPD-223537)
Supplement: Supplementary Material [file jpd-13-jpd223537-s001.pdf]

Supplementary Material

Dose Optimization of Apomorphine Sublingual Film for OFF Episodes in Parkinson's Disease: Is the Prophylactic Use of an Antiemetic Necessary?

Supplementary Table 1
Key eligibility criteria

Patient Population	Key Inclusion Criteria	Key Exclusion Criteria
New patients[a]	• Idiopathic PD by UK Brain Bank criteria<br>• Stage 1–3 by modified Hoehn and Yahr scale when ON<br>• MMSE score >25<br>• Clinically meaningful response to CD/LD with well-defined early morning OFF episodes, as determined by the investigator<br>o Receiving stable doses of CD/LD and adjunctive PD medications for ≥4 weeks (MAO-B inhibitors must be stable for ≥8 weeks) before first screening visit<br>• ≥1 OFF episode per day with total daily OFF time of ≥2 h	• Atypical or secondary parkinsonism<br>• Major psychiatric disorder<br>• Mouth cankers/sores within 30 days of the first screening visit<br>• Previous treatment with:<br>o Deep brain stimulation<br>o Intraduodenal levodopa<br>o Continuous SC-APO infusion<br>o SC-APO within 7 days of the first screening visit<br>• Currently taking:<br>o 5-HT_3 antagonists<br>o Dopamine antagonists (excluding quetiapine or clozapine)<br>o Dopamine-depleting agents
Rollover patients[b]	• Completed a prior SL-APO study and would benefit from continued treatment[c]<br>• No major changes in concomitant PD medications from prior study	• Mouth cankers/sores within 14 days of completing a previous SL-APO study<br>• Patients were excluded from the original CTH-201 (NCT03187301) study if they experienced nausea associated with the use of dopamine agonists that required treatment with an antiemetic

[a]New patients had no prior exposure to SL-APO; [b]Rollover patients had completed a prior SL-APO study (NCT03187301, NCT03292016, NCT02469090, or NCT03391882); [c]Based on investigator opinion. 5-HT_3, 5-hydroxytryptamine type 3; CD/LD, carbidopa/levodopa; MAO-B, monoamine oxidase-B; MMSE, Mini-Mental State Examination; PD, Parkinson's disease; SC-APO, subcutaneous apomorphine; SL-APO, apomorphine sublingual film.

Supplementary Table 2

Antiemetic use during dose optimization and concomitant PD medications at baseline[a]

Characteristic	Overall (N=449)	US (*n*=391)	Outside US (*n*=58)
Antiemetic use, *n* (%)[b]	253 (56.3)	210 (53.7)	43 (74.1)
Domperidone	43 (17.0)	0	43 (100)
Trimethobenzamide	207 (81.8)	207 (98.6)	0
Domperidone/trimethobenzamide[c]	3 (1.2)	3 (1.4)	0
Concomitant PD medications, *n* (%)[d]			
Dopamine agonists	271 (60.4)	221 (56.5)	50 (86.2)
MAO-B inhibitors	202 (45.0)	169 (43.2)	33 (56.9)
Amantadine	100 (22.3)	87 (22.3)	13 (22.4)

[a]Titration population (all patients who enrolled in the study and received ≥1 dose of study medication during the titration phase); [b]Patients used an antiemetic during dose optimization; [c]Patients used domperidone and trimethobenzamide during dose optimization but details regarding timing of use for each agent is unknown; [d]Defined as medication use overlapping with the first dose of SL-APO during dose optimization. MAO-B, monoamine oxidase-B; PD, Parkinson's disease; SL-APO, apomorphine sublingual film.

Supplementary Table 3

Demographics and baseline disease characteristics of new patients enrolled after prophylactic antiemetic use was made optional and who did and did not use an antiemetic[a]

Characteristic	Used an Antiemetic[b] (*n*=34)	Did Not Use an Antiemetic[b] (*n*=154)	Overall (*n*=188)
Age, y, mean (SD)	63.0 (10.8)	64.5 (8.3)	64.3 (8.8)
Male, *n* (%)	18 (52.9)	103 (66.9)	121 (64.4)
Race, *n* (%)			
White	34 (100)	147 (95.5)	181 (96.3)
Black or African American	0	5 (3.2)	5 (2.7)
Other	0	2 (1.3)	2 (1.1)
Weight, kg, mean (SD)	81.8 (19.7)	81.4 (16.4)	81.5 (17.0)
Time since PD diagnosis, y, mean (SD)	8.2 (5.3)	8.3 (4.8)	8.3 (4.9)
Time since motor fluctuations, y, mean (SD)	4.6 (3.6)	4.5 (4.0)	4.5 (3.9)
Modified Hoehn and Yahr stage when ON, *n* (%)			
0	0	1 (0.6)	1 (0.5)
1 or 1.5	2 (5.9)	7 (4.5)	9 (4.8)
2 or 2.5	26 (76.5)	131 (85.1)	157 (83.5)
3	4 (11.8)	12 (7.8)	16 (8.5)
4	0	1 (0.6)	1 (0.5)
Missing	2 (5.9)	2 (1.3)	4 (2.1)
Number of OFF episodes per day, mean (SD)	4.3 (1.9)	4.1 (1.4)	4.1 (1.5)
Total daily levodopa dose, mg, median	800.0	837.5	800.0
Concomitant PD medications, *n* (%)[c]			
Dopamine agonists	12 (35.3)	91 (59.1)	103 (54.8)
MAO-B inhibitors	16 (47.1)	70 (45.5)	86 (45.7)
Amantadine	4 (11.8)	36 (23.4)	40 (21.3)

[a]Safety population (all patients who enrolled in the study and received ≥1 dose of study medication; [b]Population was comprised entirely of US patients and therefore trimethobenzamide was the only antiemetic available for use; [c]Defined as medication use overlapping with first dose of SL-APO during dose optimization. MAO-B, monoamine oxidase-B; PD, Parkinson's disease; SL-APO, apomorphine sublingual film.

Supplementary Table 4

TEAEs occurring in ≥2% of patients overall in the dose-optimization phase[a]

Parameter, *n* (%)	Used an Antiemetic (*n*=253)	Did Not Use an Antiemetic (*n*=196)	Overall (N=449)
Overall	143 (56.5)	89 (45.4)	232 (51.7)
Yawning	26 (10.3)	17 (8.7)	43 (9.6)
Somnolence	16 (6.3)	14 (7.1)	30 (6.7)
Dizziness	16 (6.3)	11 (5.6)	27 (6.0)
Headache	19 (7.5)	4 (2.0)	23 (5.1)
Fatigue	14 (5.5)	4 (2.0)	18 (4.0)
Oral mucosal erythema	12 (4.7)	5 (2.6)	17 (3.8)
Orthostatic hypertension	7 (2.8)	6 (3.1)	13 (2.9)
Dyskinesia	5 (2.0)	7 (3.6)	12 (2.7)
Hypertension	8 (3.2)	3 (1.5)	11 (2.4)

[a]Dose-optimization population (all patients who enrolled in the study and received ≥1 dose of study medication during the dose-optimization phase). TEAE, treatment-emergent adverse event.

CTH-301 Study Investigators

Name	Institution	City	State/Province	Country
Ernest Balaguer Martinez	Hospital Universitari General de Catalunya		Sant Cugat del Vallés	Spain
Victor Biton	Clinical Trials, Inc.	Little Rock	Arkansas	United States
Ivan Bodis-Wollner	SUNY Downstate Medical Center	Brooklyn	New York	United States
Stephen Butterworth	Leeds Teaching Hospitals NHS Trust		Leeds	United Kingdom
Camille Carroll	Plymouth University		Plymouth	United Kingdom
Christine Cooper	Medical University of South Carolina	Charleston	South Carolina	United States
Andres Deik	Parkinson's Disease and Movement Disorders Center	Philadelphia	Pennsylvania	United States
Maria Francesca De Pandis	Centro Ricerche San Raffaele		Cassino	Italy
Aaron L. Ellenbogen	QUEST Research Institute	Farmington Hills	Michigan	United States
Lawrence Elmer	UT Gardner-McMaster Parkinson's Center	Toledo	Ohio	United States
Alberto J. Espay	University of Cincinnati	Cincinnati	Ohio	United States
Virgilio Gerald Evidente	Movement Disorders Center of AZ	Scottsdale	Arizona	United States
Stewart A. Factor	Emory University	Atlanta	Georgia	United States
Susan Fox	UHN Toronto Western Hospital	Toronto	Ontario	Canada
Ramon A. Gil	Parkinson's Disease Treatment Center of SW Florida	Port Charlotte	Florida	United States
Christopher G. Goetz	Rush University Medical Center	Chicago	Illinois	United States
Ira Goodman	Compass Research, LLC	Orlando	Florida	United States
John Goudreau	Michigan State University – Dept. of Neurology	East Lansing	Michigan	United States
Alida Griffith	Evergreen Health	Kirkland	Washington	United States
Donald Grosset	Queen Elizabeth University Hospital		Glasgow	United Kingdom
Robert A. Hauser	University of South Florida	Tampa	Florida	United States
Neal Hermanowicz	Irvine Health	Irvine	California	United States
Jennifer S. Hui	Keck School of Medicine at University of Southern California	Los Angeles	California	United States
Stuart H. Isaacson	Parkinson's Disease and Movement Disorders Center of Boca Raton	Boca Raton	Florida	United States
Ilia Itin	Cleveland Clinic	Cleveland	Ohio	United States
Jan Rainer Kassubek	Universitätsklinikum Ulm Neurologisches Studienzentrum im RKU		Ulm	Germany
Kevin Klos	The Movement Disorder Clinic of Oklahoma	Tulsa	Oklahoma	United States
Jaime Kulisevsky Bojarski	Hospital de la Santa Creu i Sant Pau		Barcelona	Spain
Valentina Leta	Kings College, The Maurice Wohl Neuroscience Institute		London	United Kingdom
Peter Lewitt	Henry Ford Hospital	West Bloomfield	Michigan	United States
Tsao-Wei Liang	Jefferson University Hospital Philadelphia	Philadelphia	Pennsylvania	United States

Aikaterini Markopoulou	NorthShore Neurological Institute B043D	Glenview	Illinois	United States
Maria Jose Marti	Hospital Clinic de Barcelona		Barcelona	Spain
Martha McGraw	Central DuPage Hospital - Neurodegenerative Clinic - Movement Disorders Center	Winfield	Illinois	United States
Shyamal H. Mehta	Mayo Clinic	Scottsdale	Arizona	United States
Sophie Molloy	Imperial College Healthcare Trust NHS<br>Charing Cross Hospital		London	United Kingdom
John Morgan	GRU Movement Disorders	Augusta	Georgia	United States
Siegfried Muhlack	St. Josef-Hospital, Klinikum der Ruhr-Universitaet-Bochum, Neurologische Klinik		Bochum	Germany
Anthony Nicholas	University of Alabama at Birmingham	Birmingham	Alabama	United States
William Ondo	Houston Methodist Neurological Institute	Houston	Texas	United States
Marco Onofrj	Aging Research Center, Ce.S.I. University Foundation, Chieti-Pescara<br>Behavioural Neurology & Movement Disorders Unit		Chieti	Italy
Fernando Pagan	MedStar Georgetown University Hospital	Washington	District of Columbia	United States
Rajesh Pahwa	University of Kansas Medical Center	Kansas City	Kansas	United States
Suvankar Pal	Forth Valley Royal Hospital		Larbert	United Kingdom
Sotirios Parashos	Park Nicolet Institute – Stuthers Parkinson’s Center	Golden Valley	Minnesota	United States
Ariane Park	The Ohio State University Wexner Medical Center	Columbus	Ohio	United States
Nicola Pavese	Newcastle University,<br>Clinical Ageing Research Unit		Newcastle upon Tyne	United Kingdom
Elizabeth Peckham	Central Texas Neurology Consultants	Round Rock	Texas	United States
Walter Pirker	Wilhelminenspital		Vienna	Austria
George Plotkin	East Texas Medical Center	Tyler	Texas	United States
Werner Poewe	Medical University Innsbruck Neurology Department		Innsbruck	Austria
Olivier Rascol	Centre d'Investigation Clinique, CIC 1436, CHU Purpan		Toulouse	France
Jason Raw	Fairfield General Hospital		Bury	United Kingdom
Stephen Reich	University of Maryland	Baltimore	Maryland	United States
George Ricaurte	Johns Hopkins University	Baltimore	Maryland	United States
Susie Ro	Swedish Neuroscience Research	Seattle	Washington	United States
Bradley J. Robottom	Raleigh Neurology Associates, P.A.	Raleigh	North Carolina	United States
Johannes Schwarz	Kliniken Kreis Mühldorf a. Inn Klinik Haag i.OB		Haag	Germany
Burton Scott	Duke University – Movement Disorders Clinic	Durham	North Carolina	United States

Lauren Seeberger	University of Colorado School of Medicine	Aurora	Colorado	United States
Binit Shah	University of Virginia Adult Neurology	Charlottesville	Virginia	United States
Ray Sheridan	Royal Devon & Exeter NHS Foundation Trust		Exeter	United Kingdom
Holly Shill	Muhammed Ali Parkinson and Movement Disorder Center/Barrow Neurological Institute	Phoenix	Arizona	United States
Mustafa Siddiqui	Wake Forest Medical Center	Winston-Salem	North Carolina	United States
Monty Silverdale	Manchester University		Salford	United Kingdom
Carlos Singer	University of Miami, Miller School of Medicine	Miami	Florida	United States
John T. Slevin	University of Kentucky	Lexington	Kentucky	United States
Fabrizio Stocchi	IRCCS San Raffaele Pisana – Clinical Trial Center		Rome	Italy
Karen Thomas	Sentara Neuroscience Institute	Virginia Beach	Virginia	United States
Daniel D. Truong	The Parkinson's & Movement Disorder Institute	Fountain Valley	California	United States
Winona Tse	Bendheim Parkinson's and Movement Disorder Center (Mount Sinai Medical Center)	New York	New York	United States
Ergun Uc	University of Iowa Dept. of Neurology	Iowa City	Iowa	United States
Alberto Vasquez	Suncoast Neuroscience Associates Inc.	St. Petersburg	Florida	United States
Cheryl Waters	Columbia University Medical Center - Neurological Institute, Movement Disorders	New York	New York	United States
Cindy Zadikoff	Northwestern University	Chicago	Illinois	United States

Supplementary Figure 1. Study design

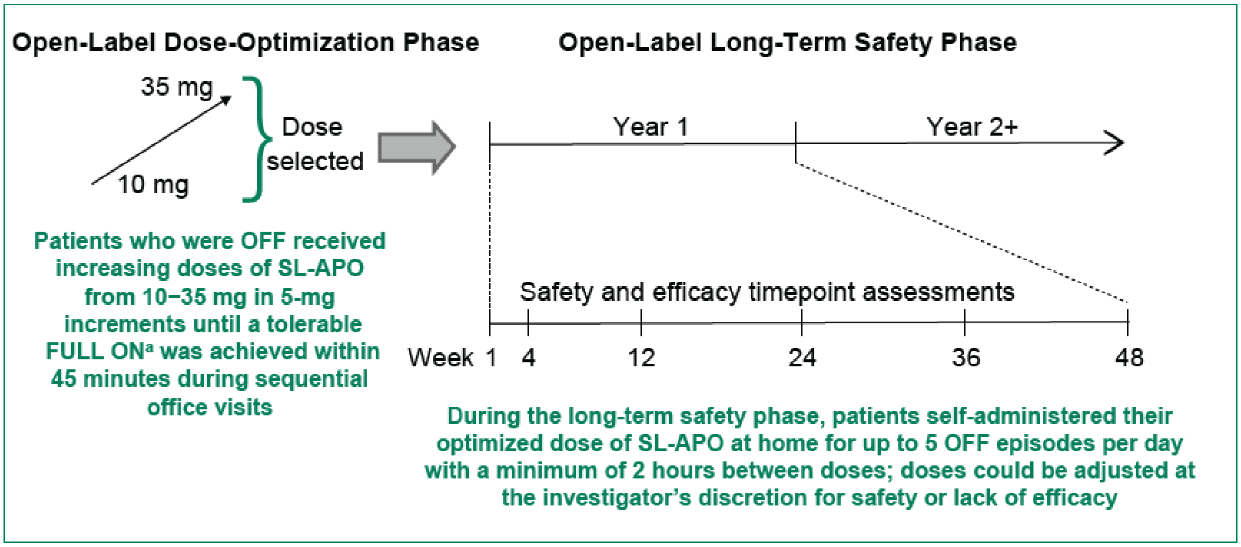

[a]FULL ON was patient- and investigator-assessed and defined as response that provided the patient with benefit related to mobility, stiffness, and slowness; adequate motor function to perform normal daily activities; and a response that was comparable to or better than the normal response to their Parkinson's disease medications before enrolling in the study.

SL-APO, apomorphine sublingual film.
